# Targeting matrix metalloproteases: A promising strategy for herbal medicines to treat rheumatoid arthritis

**DOI:** 10.3389/fimmu.2022.1046810

**Published:** 2022-11-09

**Authors:** Ruo-Lan Li, Hu-Xinyue Duan, Qi Liang, Yong-Liang Huang, Ling-Yu Wang, Qing Zhang, Chun-Jie Wu, Shu-Qin Liu, Wei Peng

**Affiliations:** ^1^ State Key Laboratory of Southwestern Chinese Medicine Resources, School of Pharmacy, Chengdu University of Traditional Chinese Medicine, Chengdu, China; ^2^ Hospital of Chengdu University of Traditional Chinese Medicine, Chengdu, China

**Keywords:** matrix metalloproteinases, rheumatoid arthritis, herbal medicines, therapeutic, strategy

## Abstract

As a type of metalloproteinase, matrix metalloproteinases (MMPs) can be divided into collagenase, gelatinase, stromelysins, membrane-type (MT)-MMPs and heterogeneous subgroups according to their structure and function. MMP contents in the human body are strictly regulated, and their synthesis, activation and inhibition processes should be kept in a certain balance; otherwise, this would result in the occurrence of various diseases. Rheumatoid arthritis (RA) is a known immune-mediated systemic inflammatory disease that is affected by a variety of endogenous and exogenous factors. In RA development, MMPs act as important mediators of inflammation and participate in the degradation of extracellular matrix substrates and digestion of fibrillar collagens, leading to the destruction of joint structures. Interestingly, increasing evidence has suggested that herbal medicines have many advantages in RA due to their multitarget properties. In this paper, literature was obtained through electronic databases, including the Web of Science, PubMed, Google Scholar, Springer, and CNKI (Chinese). After classification and analysis, herbal medicines were found to inhibit the inflammatory process of RA by regulating MMPs and protecting joint structures. However, further preclinical and clinical studies are needed to support this view before these herbal medicines can be developed into drugs with actual application to the disease.

## 1 Introduction

Rheumatoid arthritis (RA) is an immune-mediated systemic inflammatory disease that is affected by a variety of endogenous and exogenous factors, and is characterized by synovial hyperplasia and progressive joint destruction (1). The prevalence of RA in the population is approximately 0.5-1%. The incidence of RA peaks between the ages of 40 and 60 years, and its prevalence is significantly higher in women than in men (2). In existing studies, it is generally believed that RA is a rare and nonfatal disease. However, during development of the disease, joint tissues, including cartilage and bone, experience nonnegligible damage, which can seriously affect the life quality and could even reduce the life expectancy of patients (3, 4). Unfortunately, the pathogenesis of RA has not been fully elucidated up to now, which brings great challenges to the cure of RA (5). Retrospective studies on the pathogenesis of RA have found that genetic and environmental factors may be important inducers of RA (6, 7). Among the current available treatment modalities, nonsteroidal anti-inflammatory drugs (NSAIDs) are commonly used to suppress inflammation and relieve pain, while glucocorticoids are used to prevent long-term joint erosion (8). In addition, disease-modifying anti-rheumatic drugs (DMARDs), which exert anti-inflammatory and immunomodulatory effects through different pharmacological mechanisms, are often used as mainstay treatments in newly diagnosed RA cases. It is worth noting that since DMARDs have no direct anti-inflammatory or analgesic effects, there are no immediate effects (9). At the same time, biological agents that can selectively inhibit some specific molecules in the immune system have gradually been applied to RA (9). In RA treatments, high doses of drugs are often used to enable drugs to reach diseased joints and exert their curative effects, which are accompanied by toxicity and side effects. For example, NSAIDs and conventional DMARDs have apparent gastrointestinal and hepatorenal toxicity (10). Glucocorticoids can cause adverse reactions including osteoporosis, hypertension, and hyperglycaemia, while biologics may lead to autoimmune syndromes (11). Therefore, the development of new adequate pharmaceutical preparations is of great significance for conquering RA diseases.

To date, researchers have identified approximately 600 proteases in humans, including endopeptidases and exopeptidases (12). They are an indispensable part of life due to their strict regulation of various physiological processes in the human body, including autophagy, protein degradation, cell death, immune response and signal transduction (13, 14). In contrast, when protease activities are out of balance, this will result in many diseases (15). As a type of endopeptidase, metalloproteinases are related to extracellular pathways and can participate in the hydrolysis of the internal peptide bonds of polypeptide chains. Among them, matrix metalloproteinases (MMPs), as the most important metalloproteinases, are involved in the pathogenesis of various diseases, including RA (16). Synovial joint lesions are often accompanied by abnormally elevated MMP levels, suggesting that MMPs are closely related to the development of RA. This conclusion has been continuously confirmed in recent decades (16, 17). Meanwhile, with the deepening of research, we have been able to determine that MMPs are mainly responsible for the irreversible destruction of cartilage, bone and tendons in joints. Moreover, RA can be partially relieved after the use of tissue inhibitors of MMPs (TIMPs) (18). Therefore, MMPs can be considered important therapeutic targets for RA. Furthermore, increasing evidence has suggested that herbal medicines have many advantages in treating RA. They have promising roles in improving RA by participating in multiple pathways such as immune regulation, the inflammatory response, and angiogenesis (19). In this review, we focused on the regulation of MMPs by herbal medicines in RA to contribute to the development of new therapeutic drugs targeting MMPs in RA. Information on regulation of MMPs and treatment of RA by herbal medicines through MMPs was obtained through electronic database searches, including the Web of Science, PubMed, Google Scholar, Springer, and CNKI (Chinese). “Herbal medicine”, “matrix metalloproteinases” and “rheumatoid arthritis” were used for keyword screening, and the searched literature was classified and managed.

## 2 Classification and structure of MMPs

**Figure 1:**
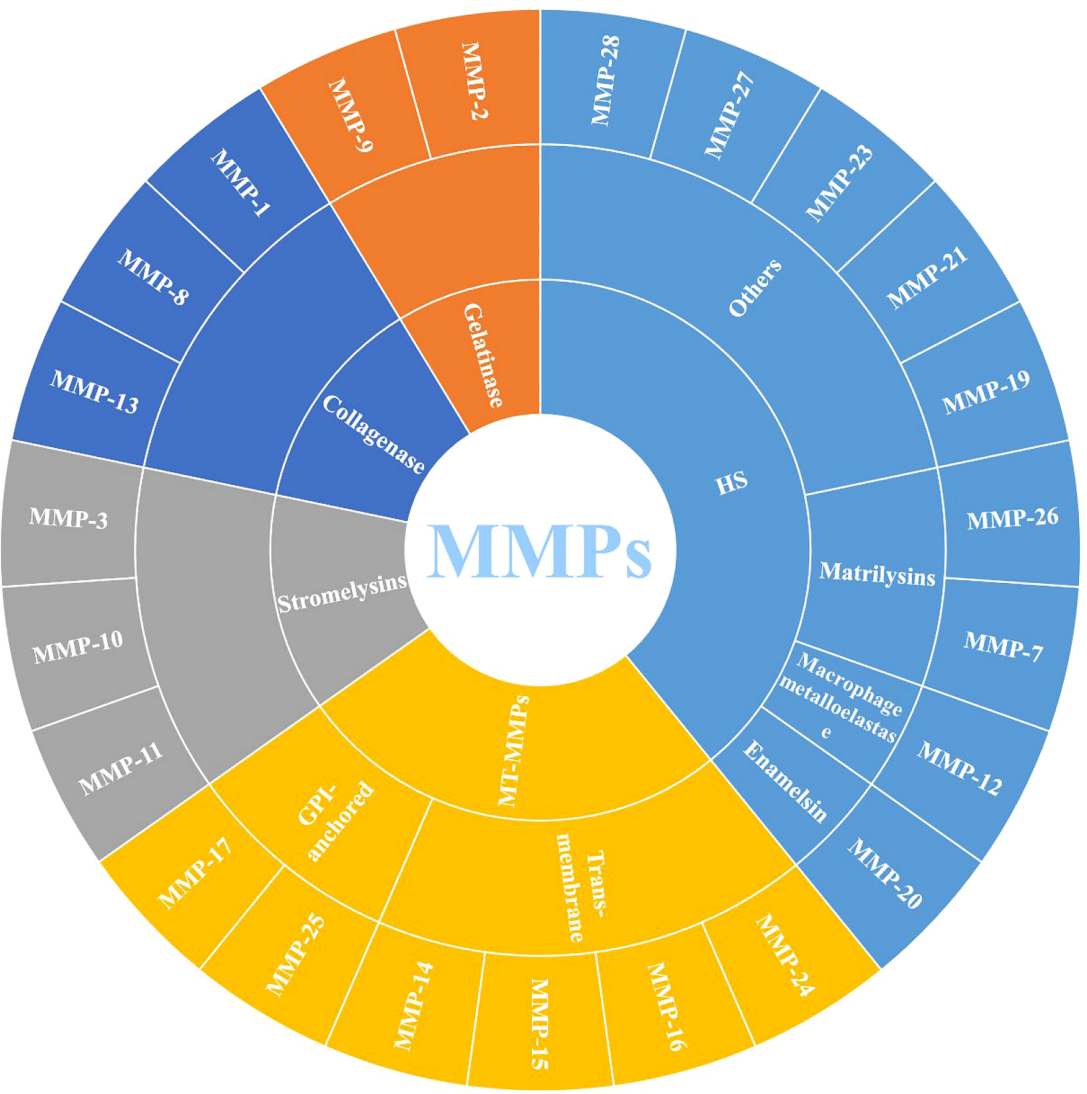
Classification of MMPs (MMPs: Matrix metalloproteinases; MT-MMPs: Membrane-type MMPs, and HS: Heterogeneous subgroups).

MMPs are zinc-dependent proteolytic enzymes, that can participate in various physiological and pathological processes, such as extracellular matrix remodeling, cell migration and angiogenesis, and are well-known extracellular modulators (20). MMPs belong to the metzincin clan of metalloendopeptidases along with ADAM (a disintegrin and a metalloproteinase) and ADAMTS (a disintegrin and metalloproteinase with a thrombospondin motif), which contain zinc at the catalytic site for the hydrolysis of peptide bonds (21). **Figure 1** shows that 23 different MMPs have been found in humans, which can be roughly divided into 5 categories according to their different functions and structures: (1) collagenase (MMP-1, MMP-8 and MMP-13); (2) gelatinase (MMP-2 and MMP-9); (3) stromelysins (MMP-3, MMP-10 and MMP-11); (4) membrane-type (MT)-MMPs, which can be divided into two types: transmembrane-types (MMP-14, MMP-15, MMP-16 and MMP-24, also known as MT1-MMP, MT2-MMP, MT3-MMP and MT5-MMP) and glycosylphosphatidylinositol (GPI)-anchored types (MMP-17 and MMP-25, also known as MT4-MMP and MT6-MMP) and (5) heterogeneous subgroups (HS), such as matrilysins (MMP-7 and MMP-26), enamelsin (MMP-20), macrophage metalloelastase (MMP-12) and others (MMP-19, MMP-21, MMP-23, MMP-27 and MMP-28) (6, 22).

**Figure 2:**
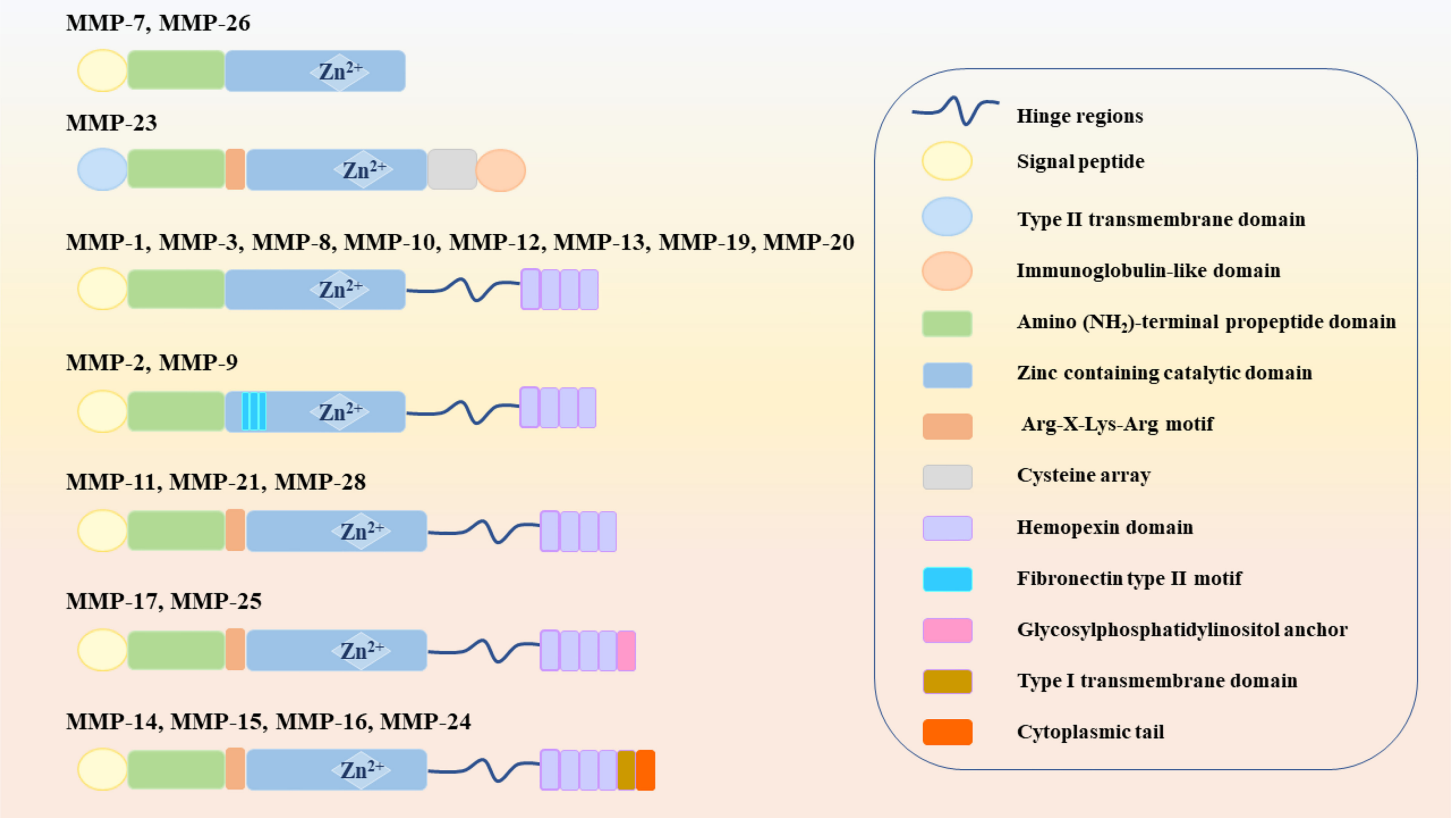
Structure of MMPs (MMPs: Matrix metalloproteinases).

Structurally, MMPs have similar components, including signal peptide (SP), amino (NH2)-terminal propeptide domains (Pro) and zinc-containing catalytic domains, which are shown in **Figure 2**. When MMPs migrate to the endoplasmic reticulum, signal peptidases cleaves SP. This basic structure is commonly found in MMP-7 and MMP-26, which also make them the smallest MMPs. In MMP-23, the type II transmembrane domain replaces the SP, making it a type II transmembrane protein. Meanwhile, the cysteine residues in Pro interact with zinc ions to inactivate MMP-23. However, there is also an Arg-X-Lys-Arg motif at the C-terminal of Pro, which can be recognized and cleaved by the pro-protein convertase furin to activate MMP-23. The catalytic domain of MMP-23 is followed by a cysteine array and an immunoglobulin-like domain (6). Except for the above MMPs, other MMPs, such as MMP-1, MMP-3, MMP-8, MMP-10, MMP-12, MMP-13, MMP-19 and MMP-20, also contain the hinge region and hemopexin (Hpx) C-terminal domain. The hinge region connects the Hpx domain to the catalytic domain. Among them, the catalytic substrate diversity is determined by the inclusion of four Hpx-like repeats in the Hpx domain. Meanwhile, the degradation of collagen and gelatin by MMP-2 and MMP-9 is due to the presence of three repeats of the fibronectin type II motif in the catalytic domain. The Pro of MMP-11, MMP-21 and MMP-28 also contain cysteine residues and Arg-X-Lys-Arg motifs. MT-MMPs, on the other hand, connect to the type I transmembrane domain or glycosylphosphatidylinositol (GPI) anchor after the Hgx domain on the basis of the MMP-11 structure (23).

## 3 Regulation of MMPs

To maintain a balance between anabolism and catabolism of joint tissues, the synthesis, activation and inhibition of MMPs are strictly regulated (24). In existing studies, it has been found that regulation of MMPs is mainly achieved by regulation of transcriptional and posttranscriptional activities.

Genes encoding MMPs are mainly expressed in connective tissue fibroblasts but also in monocytes, macrophages, endothelial cells and neutrophils. In normal tissues, MMP expression are maintained at constant low levels, while under pathological conditions such as RA, MMP expression increase sharply (25). The mechanisms that regulate the transcription of MMPs are extremely complex. Among them, the activator protein 1(AP-1) binding site at 73 bp or 1602 bp is a key regulator of MMP transcription, which can be activated by Jun and Fos family transcription factor proteins to form c-Jun/c-Jun homodimers or c-fos/c-Jun heterodimers, which in turn induce transcription of MMPs (26). Meanwhile, in MMP-1 and MMP-13, AP-1 cooperates with polyomavirus enhancer activator-3 (PEA3) to participate in the transcriptional activation of MMPs (26). Gene transcription of MMPs can also be induced by proinflammatory cytokines (e.g., IL-Iβ and TNF), growth factors (including epidermal growth factor (EGF), platelet-derived growth factor (PDGF), basic fibroblast growth factor (bFGF) and transforming growth factor β (TGF-β)), among which, TGF-β can promote or inhibit transcription of MMPs in different cell types or cell states (27). In addition, many signaling pathways can also regulate the gene expression of specific MMPs through signal transduction pathways. For example, activation of NF-κB leads to transcriptional activation of MMP-1, MMP-3 and MMP-9, while MAPK, JNK and p38 can promote transcription of MMPs by increasing AP-1 levels (28). Notably, in some MMPs, such as MMP-1, MMP-3 and MMP-13, due to the presence of the AUUUA sequence in their 3’ untranslated region genes, the posttranscriptional mRNA half-lives are extremely short, thus ensuring that MMPs can be maintained at low levels (29).

**Figure 3:**
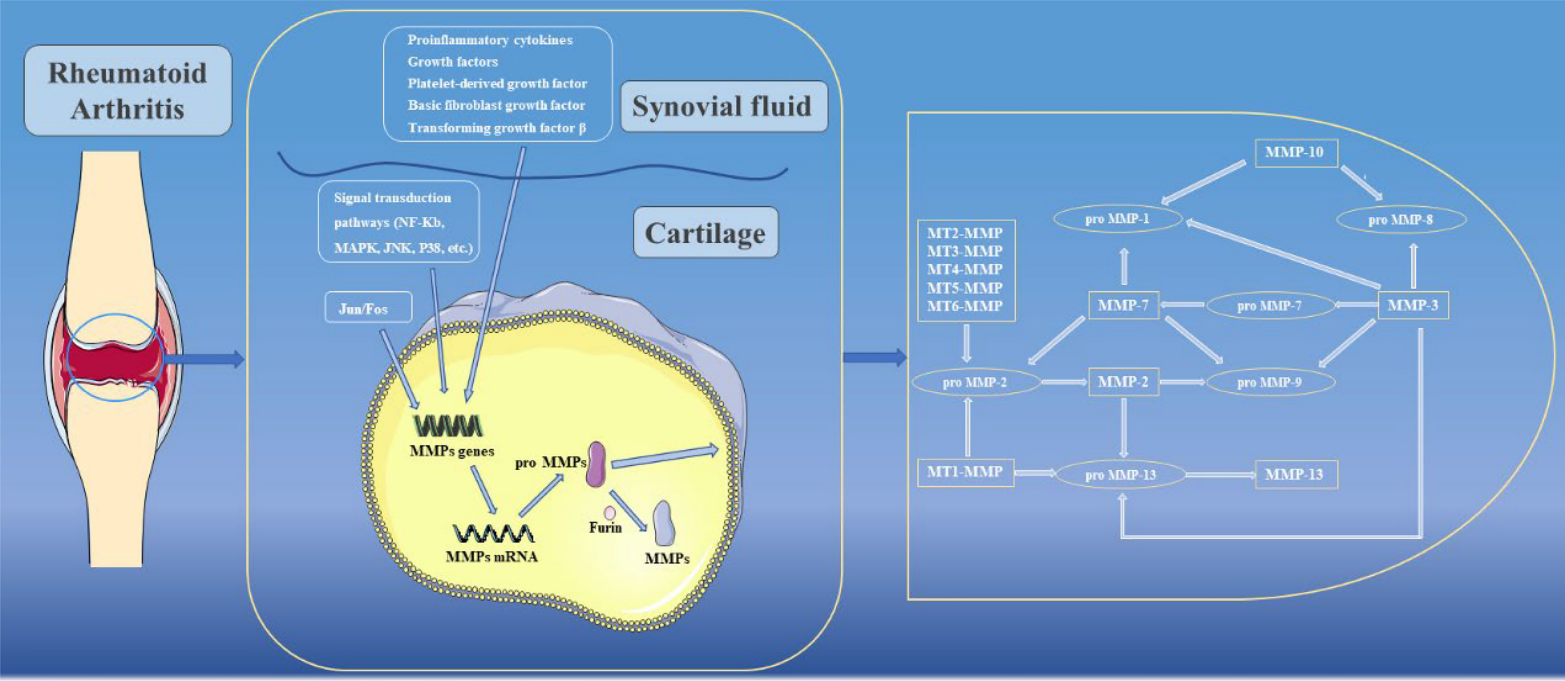
Regulation of MMPs and enzyme activation relationships between MMPs.

When MMPs are synthesized, the cysteine residues located in Pro need to be removed to become active. In the present study, activation of MMPs can be broadly divided into two pathways, namely intracellular activation and extracellular activation. In the intracellular pathway, Arg-X-Lys-Arg motifs can be recognized and activated by furin in the Golgi apparatus, and then transferred to the cell surface to activate other MMPs (30). In contrast, the extracellular pathway mainly occurs on the surfaces of cell membranes or in tissues. Among them, MMP-2 and MMP-13 are mainly activated by activated MT-MMPs on cell membrane surfaces (31). In tissues, a variety of enzymes are involved in activating MMPs, including cathepsin B and plasminogen activator urokinase type (uPA) (32, 33). In addition, some active MMPs in tissues are the activating enzymes of other MMPs. For example, MMP-10 can activate pro-MMP-8, while MMP-13 can activate pro-MMP-9, and other enzyme activation relationships are shown in **Figure 3** (34). In addition to the above processes, there are endogenous inhibitors in the body that can block the activity of MMPs. Among them, TIMPs are closely related to joints. To date, researchers have identified four TIMPs, among which TIMP-2 can inhibit the activation process of MMPs and activity of MMPs after activation (35). In addition to its MMP inhibitory activity, TIMP-3 has a wide range of inhibitory effects. For example, TIMP-3 can inhibit the activity of ADAM-17 (also known as TNF-α converting enzyme, TACE) and ADAMTS-4 and -5 (aggrecan enzymes) (36–38). However, the activity of TIMPs remains controversial in some current studies, and these results showed that although TIMPs were able to reduce the effect of MMPs, their active effects were low. When TIMPs are overexpressed, they can also promote cell invasion and apoptosis, thereby promoting the development of RA (39–41). In addition, clinical attempts to use exogenous TIMPs to inhibit MMPs have generally failed (42).

## 4 The role of MMPs in RA

### 4.1. MMPs act as mediators of inflammation

RA is an autoimmune disease that is associated with a chronic inflammatory process that affects multiple joints throughout the body. Although many molecular mechanisms have been used to explain the pathogenesis of RA, the exact etiology of RA is not well understood. It is generally accepted that genetic susceptibility and some stimulating events can induce an initial immune or inflammatory response in joints. Subsequently, inflammatory cells, which consist of neutrophils and macrophages, are recruited into the joints and release large amounts of inflammatory cytokines, such as IL-Iβ, TNF (also known as TNF-α), IL-6, and CXCL8. On the one hand, the released inflammatory cytokines can promote the proliferation of synovial fibroblasts, recruit macrophages and immune cells to form pannus and jointly invade and destroy cartilage, tendon and bone. On the other hand, they can further induce migration of inflammatory cells to the joints and aggravate immune and inflammatory responses in the body (43, 44). In addition, activated inflammatory cells and cytokines can also induce expression and secretion of MMPs, which in turn affect the actions of chemokines and cytokines. However, previous studies have shown that MMPs may promote or inhibit inflammation through different effects. For example, MMP-1, MMP-2, MMP-13 and MMP-14 can inactivate chemokines by cleaving monocyte chemoattractant protein-3 (MCP-3), thereby losing their ability to recruit monocytes and leukocytes (45, 46). At the same time, when MMP-8 expressions were restricted, neutrophil infiltration increased and RA manifestations were aggravated in mice (47). In contrast, MMP-7 can increase infiltration of inflammatory cells and promote the inflammatory response by shedding the ectodomain of syndecan-1 (48). Similarly, overexpression of MMP-12 in macrophages significantly enhanced the inflammatory response in RA, which was accompanied by increased synovial infiltration (49). Therefore, MMPs have dual roles in inflammation, and the balance between proinflammatory and anti-inflammatory signaling needs to be tightly regulated.

### 4.2. MMPs destroy joint structure

Complete joints consist of articular bones, articular cartilage, fibrous capsules, and synovial membrane. Articular cartilage is attached to the apex of the contact surface of two or more articular bones, and there is no vascular distribution. Synovial membranes can secrete lubricating synovial fluid and provide nutrients to cartilage through microcirculation. During the development of RA, in addition to the inflammatory response, RA is characterized by pathological changes in synovial tissue, cartilage and bone (50, 51). Previous studies have confirmed that activated osteoclasts can change the local environment to an acidic pH while secreting cathepsin K, which can degrade bone in an acidic environment. MMP activities are inhibited in acidic environments and therefore have no apparent effect on the calcified bone matrix (52). However, in RA, degradation of the cartilage matrix is largely attributed to MMPs. In existing studies, the expressions of MMP-1, MMP-2, MMP-3, MMP-7, MMP-8, MMP-9, MMP-13 and MT1-MMP are closely related to RA.

Healthy articular cartilage is mainly composed of collagen and proteoglycan aggregates. Among them, a single aggregate of proteoglycan aggregates consists of approximately 100 core protein units called aggrecans. It is well known that aggrecans possess three globular domains (e.g., G1, G2, and G3) and can covalently bind to negatively charged glycosaminoglycans (GAGs), such as chondroitin sulphate, keratan sulphate, and dermatan sulphate. Subsequently, single aggregates bind noncovalently to hyaluronic acid consisting of repeated glucuronic acid and N-acetylglucosamine disaccharides, and eventually form proteoglycan aggregates. In proteoglycan aggregates, GAGs can attract water due to their negative charge, providing cartilage protection and reducing the friction coefficients of joint surfaces (53, 54). In RA, ADAMTs are the major enzymes involved in the degradation of proteoglycan aggregates in cartilage, while only some MMPs, such as stromelysin, MMP-1, MMP-2 and MMP-3, can participate in the cleavage of proteoglycan aggregates (55, 56). When MMPs act on proteoglycans, the G1 domain dissociates from the aggregates and diffuses into the synovial fluid, thereby losing its structural function.

As another important component of articular cartilage, collagen is also the most abundant protein in the animal kingdom. In articular cartilage, type II collagen is the most common collagen, while type IX and XI collagen are only a minority. Notably, type II collagen is a unique component of cartilage, consisting of three Alpha1 (type II) chains that support the rigid structure of cartilage (57). In organisms, collagen degradation is almost exclusively mediated by MMPs. First, collagenase (e.g., MMP-1, MMP-8 and MMP-13) can target the collagen triple helix between Gly775 and Leu776 to cleave collagen, producing a 1/4 C-terminus and 3/4 N-terminus (56, 58). Subsequently, these deformed molecules are further degraded by gelatinases (MMP-2 and MMP-9) (59). Although all three collagenases can cleave the triple helix structure of collagen and due to their different locations and substrate preferences, the degradation effects of these three collagenases are also different. Among the three collagenases, MMP-1 and MMP-8 are localized to the superficial surfaces of cartilage. MMP-1 is stably expressed in a variety of cells and has a certain ability to cleave type I, II, III, VII and X collagens, while MMP-8 can cleave type I, II and III collagens. Notably, MMP-8 is mainly stored in neutrophils, and its activity is highest when cleaving type I collagen (60, 61). In addition, MMP-13, as the main force in the cleavage of type II collagen, is mainly distributed in chondrocytes, and its activity in cleavage of type II collagen is 5-10 times that of MMP-1. In some studies, MT1-MMP was shown to directly degrade collagen and indirectly participate in collagen degradation through proMMP-13 activation (53).

MMPs can degrade all components of the extracellular matrix, and most MMPs can degrade a variety of extracellular matrices. Gelatinases degrade proteoglycans in addition to denatured collagen (62, 63). Stromelysins have a wide range of specificity for fibronectin, laminin, and elastin. Stromelysin-1 (MMP-3) not only can damage the specificity of proteoglycans, but also activate proMMP-1 to degrade collagen, so stromelysin-1 plays a dual role in RA (56).

In addition to the above methods, synovial tissue invasion, which is closely related to MMP expression, is another important pathway of cartilage destruction and is closely related to the inflammatory process. In addition to MMP-12, MMP-13 is also thought to be involved in the invasion of RA synovial fibroblasts (RASFs) into cartilage. Direct evidence showed that cartilage erosion was significantly alleviated after the use of a specific MMP-13 inhibitor in vivo (64). Interestingly, selective silencing of MMP-13 in an in vitro model had no apparent effect on RASF invasion (65). Moreover, MT1-MMP not only directly degrades collagen, but also promotes synovial invasion. After MT1-MMP was specifically inhibited or selectively knocked out, the invasion effect of RASF on cartilage was significantly alleviated (66, 67). In addition, MT1-MMP may contribute to cartilage destruction by promoting angiogenesis and endothelial cell migration (68).

## 5 Herbal medicines can treat RA by regulating MMP

In recent years, the existing drugs have shown certain deficiencies in treating RA, but the incidence of RA is still high. Therefore, it is urgent to find new therapeutic drugs. Herbal medicines are derived from plants that exist in nature. They have a wide range of sources and various types, and many have multitarget effects, making them excellent source of drugs.

### 5.1 Extracts from herbal medicines

#### 5.1.1 Herbal medicine formulas

Qing-Luo-Yin (QLY) is an herbal medicine formula containing four Chinese herbs, namely *Sophora flavescens* Aiton, *Phellodendron amurense* Rupr., *Sinomenium acutum* (Thunb.) Rehder & E.H.Wils. and *Dioscorea collettii* var. *hypoglauca* (Palib.) S.J.Pei & C.T.Ting. QLY is commonly used to treat RA in China and has achieved remarkable results. As early as 2002, Li et al. first used a collagen induced arthritis (CIA) model to confirm that QLY had significant effects on the arthritis index, pain, and ankle swelling in CIA rats (69). Subsequently, Li et al. continued to use the CIA model for in-depth explorations. The results showed that QLY could reverse imbalances between MMP-3 and TIMP-1 expressions in RA by inhibiting the expression of MMP-3 and promoting TIMP-1 production, thereby inhibiting angiogenesis in the synovium, and finally playing a role in improving the joint morphology of rats (70). More details are shown in **Table 1**.

**Table 1:** Herbal medicine formulas that can treat rheumatoid arthritis by regulating MMPs.

Components	Plant source	Experimental model		Effective dose	Effect	Mechanism	Ref
		In vivo	In vitro				
*Qing-Luo-Yin*	*Sophora flavescens* Aiton, *Phellodendron amurense* Rupr., *Sinomenium acutum* (Thunb.) Rehder & E.H.Wils. and *Dioscorea collettii* var. *hypoglauca* (Palib.) S.J.Pei & C.T.Ting.	CIA mice		0.3 g/kg/d	Suppressing angiogenesis	MMP-3↓, TIMP-1↑	(69, 70)
Tongbiling	*Neolitsea cassia (*L.) Kosterm*., Paeonia lactiflora* Pall., *Aconitum carmichaeli* Debeaux, *Achyranthes bidentata* Blume, *Celastrus orbiculatus* Thunb, *Wisteriopsis reticulata* (Benth.) J.Compton & Schrire	CIA mice		300 mg/kg/d	Decreasing inflammation	IL-1β↓, TNF↓, MMP-2↓, MMP-3↓, MMP-9↓, IgG2a type anti-CII antibody↓	(71)
Alkaloids of *jeevaneeya* *rasayana*	*Cyperus rotundus* L., *Boerhaavia diffusa* L., *Tribulus terrestris* L., *Curculigo Orchioides* Gaertn., *Mucuna Pruriens* (L.) DC., *Withania somnifera* (L.) Dunal, *Asparagus racemosus* Willd., *Hygrophila auriculata* (Schumach.)	FCA-induced rat		10 mg/kg/day	Decreasing inflammation	PGE_2_↓, NO↓, COX-2↓, TNF, IL-6↓, MMP-9↓	(72)
Ruteng	*Boswellia carterii* Birdw, *Tinospora sinensis* (Lour.) Merr., *Cassia obtusifolia* L, *Abelmoschus manihot* (L.) Medic, *Terminalia chebula* Retz., *Lamiophlomis rotata* (Benth.) Kudo, *Pyrethrum tatsienense* (Bur. et Franch.) Ling	CIA mice		95.0, 190.0, 285.0 mg/kg/d	Decreasing inflammation; Decreasing oxidative	MMP-1↓, MMP-3↓, MMP-13↓, TNF↓, COX-2↓, iNOS↓, IL-1β↓, IL-6↓, SOD↑, MDA↓	(73)
Fufang Shatai Heji	*Glycyrrhiza Uralensis* Fisch., *Ophiopogon japonicus* (Thunb.) Ker Gawl., *Astragalus mongholicus* Bunge, *Pseudostellaria heterophylla* (Miq.) Pax, *Adenophora triphylla* (Thunb.) A.DC., *Rehmannia glutinosa* (Gaertn.) DC., *Triticum aestivum* L, *Prunella vulgaris* L. and *Dendrobium nobile* Lindl.	CIA mice		10 mL/kg/d	Alleviating synovial hyperplasia and cartilage destruction	ADAMTS-4↓, ADAMTS-5↓, MMP-9↓, MMP-13↓	(74)

ADAMTS, a disintegrin and metalloproteinase with thrombospondin motif; CIA, collagen-induced arthritis; COX, cyclo-oxygenase; FCA, Freund’s Complete Adjuvant; IL, Interleukin-1; MMPs, matrix metalloproteinases; PGE, prostaglandin E; TIMP, tissue inhibitors of MMP; TNF, tumor necrosis factor.

Tongbiling (TBL) is a Chinese herbal formula that has anti-arthritic effects and has been used clinically for many years. It consists of *Neolitsea cassia (*L.) Kosterm*., Paeonia lactiflora* Pall., *Aconitum carmichaeli* Debeaux, *Achyranthes bidentata* Blume, *Celastrus orbiculatus* Thunb and *Wisteriopsis reticulata* (Benth.) J.Compton & Schrire. Shen et al. used CIA mice as a model to validate the therapeutic effects of TBL on RA. It was found that TBL suppressed inflammation and attenuated cartilage and bone destruction, and the underlying mechanisms were corrected by decreasing the amounts of IL-1β and TNF and reducing the expressions of MMP-2, -3, and -9 (71).

Jeevaneeya rasayana (JR) is an anti-arthritic ayurvedic polyherbal formulation that consists of *Cyperus rotundus* L., *Boerhaavia diffusa* L., *Tribulus terrestris* L., *Curculigo Orchioides* Gaertn., *Mucuna Pruriens* (L.) DC., *Withania somnifera* (L.) Dunal, *Asparagus racemosus* Willd. and *Hygrophila auriculata* (Schumach.) Heine. Shyni et al. studied the anti-arthritic effects of the alkaloid fraction of Jeevaneeya Rasayana (AJR) in an adjuvant-induced rat model and found that AJR attenuated paw oedema. Furthermore, the levels of PGE2 and serum NO, activity of COX-2 and mRNA expressions of TNF, IL-6 and MMP-9 were downregulated, suggesting that AJR has pharmacological anti-arthritis activity (72).

Compound Ruteng (CRT) is a Chinese herbal formula that has been used to treat rheumatism for centuries in the Tibetan area of China. It contains seven herbal medicines, including *Boswellia carterii* Birdw, *Tinospora sinensis* (Lour.) Merr., *Cassia obtusifolia* L, *Abelmoschus manihot* (L.) Medic, *Terminalia chebula* Retz., *Lamiophlomis rotata* (Benth.) Kudo, *Pyrethrum tatsienense* (Bur. et Franch.) Ling. To reveal the mechanisms of the anti-arthritis effect of CRT, Huang et al. used network pharmacology and experimental validation. Their results showed that CRT attenuated inflammation in paw swelling, synovial joints and cartilage in collagen-induced arthritic (CIA) rats and downregulated MMP-1, MMP-3, MMP-13, TNF, COX2 and iNOS, suggesting that CRT may exert anti-arthritis effects by inhibiting inflammatory cytokines, suppressing oxidative stress, and balancing MMPs (73).

Another herbal medicine formula, Fufang Shatai Heji (FST), also protects joint structures. It is mainly composed of *Glycyrrhiza Uralensis* Fisch., *Ophiopogon japonicus* (Thunb.) Ker Gawl., *Astragalus mongholicus* Bunge, *Pseudostellaria heterophylla* (Miq.) Pax, *Adenophora triphylla* (Thunb.) A.DC., *Rehmannia glutinosa* (Gaertn.) DC., *Triticum aestivum* L, *Prunella vulgaris* L. and *Dendrobium nobile* Lindl. First, Fan et al. found that FST could protect CIA mice from spleen injury, and they subsequently speculated that FST might also protect against cartilage injury in CIA mice. Their conjecture was verified after a tissue staining analysis of knee and ankle joints. Cartilage destruction was significantly suppressed in CIA mice after FST treatment. After further exploring the underlying mechanism, it was found that FST could inhibit collagen degradation by downregulating the expressions of MMP-9 and MMP-13 and downregulating the expressions of ADAMTS-4 and ADAMTS-5 to inhibit aggrecan degradation, and the two acted together to protect the structure of cartilage (74).

#### 5.1.2 Plant extracts

In addition to herbal medicine formulas, researchers have also explored the effects of single herbal extracts on MMP expression in RA (shown in **Table 2**). In 2001, Sylvester studied the effect of *Tripterygium wilfordii* Hook. F. (TWHF) extract on TNF/IL-1β/IL-17-induced femoral head primary chondrocytes/confluent primary bovine chondrocytes/human synovial fibroblasts and TNF-induced human chondrocytes, and found that a TWHF extract showed significant anti-inflammation activity and could inhibit cartilage matrix resorption by MMP-3 and MMP-13 by interfering with the DNA binding ability of the AP-1 and NF-kB transcription factors (75).

**Table 2:** Plant extracts that can treat rheumatoid arthritis by regulating MMP.

Components	Plant source	Experimental model		Effective dose	Effect	Mechanism	Ref
		In vivo	In vitro				
Extract of *Tripterygium wilfordii* Hook F	*Tripterygium wilfordii* Hook F		TNF/IL-1β/IL-17-induced femoral head primary chondrocytes/confluent primary bovine chondrocytes/human synovial fibroblasts	2.5 and 5 ng/mL	Decreasing inflammation; Blocking cartilage matrix resorption	MMP-3↓, MMP-13↓	(75)
			TNF-induced human chondrocytes	5 ng/mL		AP-1↓, NF-κB↓	
Ethanol extract from 12 herbs (PG201)	*Chaenomelis speciosa* Nakai, *Achyranthes bibentata* Blume, *Angelica sinensis* Oliv., *Cnidium officinale* Makino, *Gastrodia elata* Blume, *Acanthopanax senticosus* Maxim., *Carthamus tinctorius* L., *Cinnamomum aromaticum* Nees, *Gentiana macrophylla* Pall., *Ledebouriella seseloides* Wolff, *Clematis chinensis* Retz., and *Phlomis umbrosa* Turczaninow	Collagen-induced arthritis (CIA) mice		0.2 mg/d	Decreasing inflammation	TNF↓, IL-1β↓, TIMP-2↑, TIMP-2/MMP-2↑, IL-4↑	(76)
N-butanol extract of *Panax notoginseng* (Burk.) F. H. Chen (*P. notoginseng*) (BT-201)	*Panax notoginseng* (Burk.) F. H. Chen	CIA mice		15 mg/kg/d	Decreasing inflammation	TNF↓, IL-1β↓, iNO↓, MMP-13↓, p-IKKb↓, p-ERK↓, p-p38↓, p-JNK↓	(77)
			LPS-induced THP-1 cells	0.125, 0.25, 0.5 mg/mL			
Ethanol extract of *Ligularia fischeri* (Ledeb.) Turcz	*Ligularia fischeri* (Ledeb.) Turcz		IL-1β–induced SW982 cells	10 and 50 μg/ml	Decreasing inflammation	TNF↓, IL-6↓, MMP-3↓, p-JNK↓, p-p38↓, NF-κB↓, AP-1↓	(78)
Water extract of six traditional medicinal plants	*Artemisiae Capillaris* Thunb, *Phyllostachys nigra* var. *henonis* (Mitford) Rendle, *Senna tora* (L.) Roxb., *Cornus officinalis* Siebold & Zucc., *Leonurus cardiaca* L. and *Sesamum indicum* L.		IL-1β-induced FLSs	1, 10 and 100 µg/mL	Decreasing inflammation	MMP-3↓	(79)
Aflapin	*Boswellia serrata* Roxb.	Freund’s Complete Adjuvant (FCA)-induced rats		100 mg/kg/d	Decreasing inflammation	TNF↓	(80)
			IL-1β-induced HCH cells	0.25, 0.5 and 1 μg/ml	Improving cell proliferation; Improving glycosaminoglycans production		(80)
			TNF-induced SW982 cells	1 μg/ml	Inhibiting secretion of collagen degrading enzyme	MMP -3↓	(80)
Water extract of *Salacia reticulata* Wight	*Salacia reticulata* Wight		IL-1β-induced MTS-C H7 cell	50 µg/mL	Inhibiting cell proliferation	MMP-3↓, MMP-13↓	(81)
*Celastrus orbiculatus* Thunb. extract	*Celastrus orbiculatus* Thunb.		IL-1β and TNF combination-stimulated RA-FLSs	5, 10 and 20 μg/ml	Inhibiting cell invasion	MMP-9↓, p-IκBα↓, NF-κB↓	(82)
Ethyl acetate fraction from Angelica sinensis	The root of *Angelica sinensis* (Oliv.) Diels		IL-1β-induced RASFs	100 μg/mL	Inhibiting cell proliferation	MMP-1↓, MMP-3↓, COX-2↓, PGE2↓, p-ERK-1/2↓, p-p38↓, p-JNK↓, NF-κB↓	(83)
Extract of *S. plebeia*	*Salvia plebeia* R. Br.	CIA mice		2, 10, 50 mg/kg	Suppressing the development of CIA; Decreasing inflammation	MMP-1↓, MMP-3↓, IL-1β↓, IL-6↓, NF-κB↓, Akt↓	(84)
Methanol extract of *C. ternatea*	*Clitoria ternatea* Linn. *(C. ternatea)*	CIA mice		50 mg/kg/d	Decreasing inflammation; Decreasing oxidative stress	MPO activity↓, TNF↓, IL-1β↓, IFNγ↓, IL-6↓, IL-12p4↓, CXCL8↓, MCP-1↓, ROS↓, TNFR1↓, TLR2↓, iNOS↓, COX-2↓, MMP-2↓	(85)
quercetin-3ß-D-glucoside				2.5 mg/kg/d			
Ethanol extract of *Gastrodia elata* Blume	*Gastrodia elata* Blume		TNF-induced RA-FLS	1, 5 and 10 μg/ml	Decreasing inflammation	IL-6↓, CXCL8↓, MMP-3↓, MMP-13↓, p-p65↓, IkBα↑	(86)
			TNF-induced RASFs				
Extracts from *Strychnos nux-vomica* L.	*Strychnos nux-vomica* L.		SW982 cells	10 μg/ml	Inhibiting cell proliferation; Inhibiting cell migration	Wnt5a↓, Runx2↓, MMP-3↓, Bmp2↑, p-JNK↓, p- p65↓	(87)
Polyphenolic extract from extra virgin olive oil	Extra virgin olive oil		IL-1β-induced SW982 cell	12.5, 25 and 50 µg/mL	Decreasing inflammation	TNF↓, IL-6↓, COX-2↓, PGE synthase-1↓, MMP-1↓, MMP-3↓, p-JNK↓, p-p38↓, p-ERK↓, IκB-α↑	(88)
Aqueous extract of *Cinnamomi ramulus*	*Cinnamomum cassia* Presl. (Lauraceae)		TNF-induced MH7A cells	0.2, 0.4 and 0.6 mg/mL	Inhibiting cell migration and invasion	MMP-1↓, MMP-2↓, MMP-3↓,	(89)
Extractive of *Stachys inflata* var. *caucasica* Stschegl	*Stachys inflata* var. *caucasica* Stschegl.	44 women (age: 30–65 years) diagnosed with moderately active RA	Triple-blind, randomized controlled	2.4 g/day SSC + 2.4 g/day black tea 8 weeks	Reducing the number of tender, swollen joints and DAS28	HS-CRP↓, IL-1β↓, MMP-3↓	(90)

LPS, lipopolysaccharide; RA-FLSs, rheumatoid arthritis fibroblast-like synoviocytes.

In the experiment conducted by Shin et al., the researchers screened 12 herbs that may have potential therapeutic effects on RA, including *Chaenomelis speciosa* Nakai, *Achyranthes bibentata* Blume, *Angelica sinensis* Oliv., *Cnidium officinale* Makino, *Gastrodia elata* Blume, *Acanthopanax senticosus* Maxim., *Carthamus tinctorius* L., *Cinnamomum aromaticum* Nees, *Gentiana macrophylla* Pall., *Ledebouriella seseloides* Wolff, *Clematis chinensis* Retz., and *Phlomis umbrosa* Turczaninow. These herbs were extracted in 25% ethanol and then administered to CIA mice. After histopathological observation of the knee joint, it was found that articular cartilage loss was alleviated after drug treatment. At the same time, the content of TIMP-2 in the serum was significantly increased, and the ratios of TIMP-2 to MMP-2 also increased. After the detection of anti-inflammatory factors in serum, the results showed that the IL-4 levels increased after administration, while the IL-10 levels did not change significantly. In conclusion, the above extracts can affect MMP balance in vivo, inhibit inflammation and protect joint structure (76).

Chang et al. used n-butanol to extract *Panax notoginseng* (Burk.) F. H. Chen and named the extract BT-201. In vitro, BT-201 inhibited inflammation by inhibiting the NF-KB and MAPK signaling pathways, resulting in reduced secretion of TNF and IL-1β and a similar reduction in MMP-13 secretion. CIA mice were used to evaluate the efficacy of BT-201 in vivo. The results showed that the onset time of arthritis was delayed after BT-201 treatment, and cartilage destruction, bone erosion and synovial hyperplasia were alleviated, indicating that BT-201 had dual anti-inflammatory and joint structure protection effects in RA (77).

Choi et al. reported that an ethanol extract of *Ligularia fischeri* (Ledeb.) Turcz. (EELFL) decreased the amounts of TNF, IL-6 and MMP-3 in IL-1β–treated SW982 cells, which demonstrated anti-inflammatory effect of EELFL. In addition, the expressions of p-JNK, p-p38, NF-κB and AP-1 were downregulated, which could be the mechanism by which EELFL exerts anti-inflammatory effects (78).

Ra et al. used IL-1β stimulated fibroblast-like synoviocytes (FLSs) as a model to screen 35 medicinal plants in 2011. The results showed that six species, including *Artemisiae Capillaris* Thunb, *Phyllostachys nigra* var. *henonis* (Mitford) Rendle, *Senna tora* (L.) Roxb., *Cornus officinalis* Siebold & Zucc., *Leonurus cardiaca* L. and *Sesamum indicum* L., effectively inhibited the expression of MMP-3, but the pharmacodynamic effects of these six species on RA were not further investigated (79).

Aflapin contains *Boswellia serrata* Roxb. extract enriched in 3-O-acetyl-11-keto-β-boswellic acid and non-volatile oil portion. The experimental results of Sengupta et al. showed that aflapin significantly reduced the paw oedema volume of SD rats induced by Freund’s complete adjuvant (FCA) and played an anti-inflammatory role. Subsequently, the model rats were treated with drug-containing serum, and the results showed that aflapin could exert an anti-inflammatory effect by reducing the content of TNF. In IL-1β-treated human primary chondrocytes, different aflapin concentrations increased the glycosaminoglycan contents in a dose-dependent manner. In addition, aflapin also decreased the level of MMP-3 in TNF-induced SW982 human synovial cells. Taken together, these data collectively suggest that aflapin may have multiple effects in RA, both anti-inflammatory and anti-protein degradation (80).

Sekiguchi et al. used hot-water extracts of *Salacia reticulata* Wight (HSR) to explore their potential effects on RA. They obtained a synoviocyte-like cell line from mice with type II collagen antibody-induced arthritis and named it MTS-C H7. In this study, they found that HSL inhibited both IL-1β-induced cell proliferation and MMP-13 expression. At the same time, HESR was isolated and the isolated components were applied to cells, resulting in inhibition of cell proliferation by only the low molecular weight protein. Therefore, researchers hypothesized that the low molecular weight proteins in HSL may have the potential to inhibit cell proliferation and MMP expression (81).

*Celastrus orbiculatus* Thunb. (CLO) is a Chinese herb that has been widely used in folk medicine to treat inflammation. In the study of Li et al., after applying its extract to FLSs stimulated by IL-1β and TNF, it was found that CLO could inhibit the transcriptional activity of MMP-9 by inhibiting the binding activity of NF-κB in the MMP-9 promoter and the phosphorylation and nuclear translocation of NF-κB, thereby downregulating the expression and activity of MMP-9. In turn, invasion of FLSs was inhibited (82).

The roots of *Angelica sinensis* (Oliv.) Diels (AS) are a widely used herbal medicine with a protective effect on AR. Lee et al. found that the ethyl acetate fraction from Angelica sinensis (EAAS) could suppress the expression of COX-2, MMP-1 and MMP-3, decrease PGE2 production, and inhibit the activation of NF-κB and phosphorylation of MAPK pathways in IL-1β-induced RASFs, suggesting that EAAS treated RA via anti-inflammation effects (83).

*Salvia plebeia* R. Br. has also been considered in recent years for treating RA due to its excellent anti-inflammatory effects. In a 2015 study, an extract of Salvia plebeia R. Br. (ESP) was administered to CIA mice and TNF-stimulated RA synovial fibroblasts. The results showed that EPS could inhibit the expression of proinflammatory factors and MMP-3 by inhibiting the NF-κB, Akt and MAPKs signaling pathways, thereby delaying the occurrence and development of RA (84).

Adhikary et al. investigated the effects of *Niphogeton ternata* (Willd. ex Schult.) Mathias & Constance (C. ternateaand) and its main active ingredient (quercetin-3ß-D-glucoside, QG) on CIA mice, and the results revealed that C. ternateaand and QG suppressed the release of proinflammatory cytokines, chemokines and reactive oxygen species, while they could also inhibit MMP-2 expression (85).

*Gastrodia elata* Blume (GE) is a traditional Chinese herbal medicine with anti-inflammatory activity, and according to a study, GE significantly decreased the IL-6, CXCL8, MMP-3 and MMP-13 levels in TNF-induced rheumatoid arthritis fibroblast-like synoviocytes (RA-FLSs). At the same time, this decrease in inflammatory factors was accompanied by a decrease in p-p65 expression and an increase in IκBα. Therefore, GE might be a potential herbal therapy to treat RA by suppressing the inflammatory response by inhibiting the NF-κB pathway (86).

*Strychnos nux-vomica* L. (SL) has been used as medicine for thousands of years in China, and it has been gradually extended to treat RA in modern applications. In experiments by Deng et al., it was found that an alkaloid extract from SL without lappaconitine (ASLL) could significantly inhibit the proliferation and migration of SW982 cells. In a qPCR assay, ASLL showed inhibitory effects on Wnt5a, Runx2 and MMP-3 mRNA and increased the expression of Bmp2 mRNA. In addition, ASLL inhibited phosphorylation of JNK and NF-κB p65 and MMP-3 expression. In conclusion, ASLL may inhibit the proliferation and migration of FLSs by inhibiting the Wnt5A-mediated JNK and NF-κB pathways and has a certain therapeutic potential for RA (87).

Another study by Rosillo et al. reported that a polyphenolic extract (PE) from extra virgin olive oil (EVOO) showed an anti-inflammatory effect by inhibiting production of IL-6, CXCL8, MMP-3 and MMP-13, and the mechanisms might be related to suppress the phosphorylation of MAPK and the activation of NF-κB (88).

In a later study by Liu and Zhang et al., it was found that an aqueous extract of *Cinnamomi ramulus* (ACR), which was derived from the dry twigs of *Cinnamomum cassia* Presl. (Lauraceae), may have potential therapeutic effects on RA. In this study, TNF-induced RA-derived FLS MH7A cells were used as the research object. The results showed that ACR could effectively promote apoptosis of MH7A cells by increasing the expressions of BAX and caspase-3 and inhibiting the expression of Bcl-2. On the other hand, it could induce G2/M phase arrest of MH7A cells by upregulating P53, P21, cyclin D and downregulating cyclins B1, CDC2, CDK4. More importantly, ACR can inhibit the expression of MMP-1, MMP-2 and MMP-3 in MH7A cells, and effectively prevent the invasion and migration of synovial fibroblasts, thereby protecting cartilage and bone from injury (89).

In addition, a clinical trial examining the anti-arthritic effects of *Stachys inflata* var. *caucasica* Stschegl. (SS) was conducted. A triple-blind, randomized controlled clinical trial involving 44 female patients diagnosed with RA showed that SSC might decrease the number of tender and swollen joints by decreasing serum levels of IL-1β and MMP-3 (90). Other details are shown in **Table 3**.

**Table 3:** Monomers from herbal medicines that can treat rheumatoid arthritis by regulating MMP.

Family of compounds	Components	Plant source	Structure	Experimental model		Effective dose	Effect	Mechanism	Ref
				In vivo	In vitro				
Terpenoids	Triptolide	*Tripterygium wilfordii* hook f,	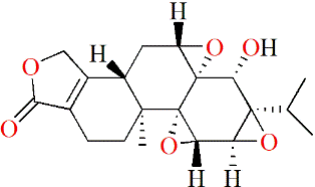		IL-1α-induced human synovial fibroblasts	28, 56 and 140 nM	Decreasing inflammation	proMMP-1↓, proMMP-3↓, TIMP-1↑, TIMP-2↑, PGE2↓, COX-2↓	(91)
					LPS-induced mouse macrophages	28 nM		IL-1α↓, IL-1β↓, TNF↓, IL-6↓
					IL-1/IL-17/TNF-induced SW1353 cells	125 and 250 nM	Decreasing inflammation	MMP-3↓, MMP-13↓, ADAMTS-4↓	(92)
	Pristimerin	Celastraceae family	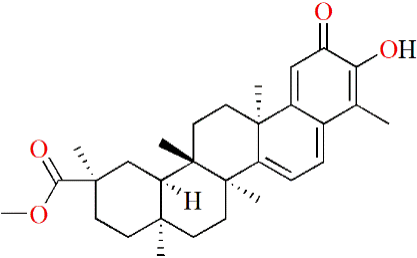	Mycobacterium tuberculosis H37Ra (Mtb)-induced rat		1 mg/kg/d	Decreasing inflammation	IL-6↓, IL-17↓, IL-18↓, IL-23↓, pSTAT3↓, ROR-γt↓, IL-10↑, IFN-γ↑,	(93)
	Swertiamarin	*Enicostema axillare* (Lam.) A. Raynal (Gentianaceae)	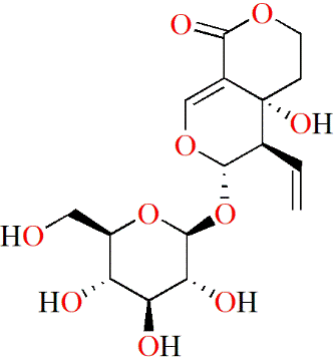	FCA-induced rats		2, 5 and 10 mg/kg/d	Inhibiting the levels of paw thickness, lysosomal enzymes; Increasing the body weight; Alleviating bone destruction	IL-1↓, TNF↓, IL-6↓, MMP-9↓, iNOS↓, PGE2↓, PPARγ↓, COX-2↓, IL-10↑, IL-4↑, p65↓, p-IκBα↓, p-JAK2↓, p-STAT3↓	(94)
				LPS-induced RAW264.7 cells	10, 25 and 50 μg/mL		p65↓, p-IκBα↓, p-JAK2↓, p-STAT3↓		
Flavonoids	Epigallocatechin-3-gallate	Tea	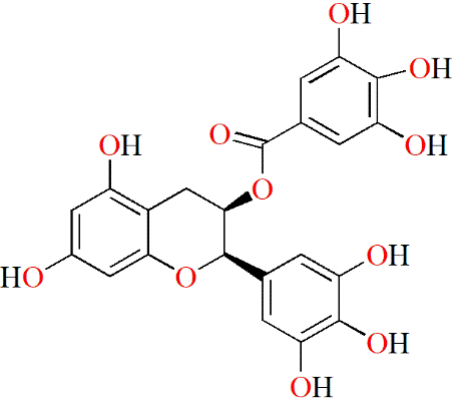		IL-1β–induced RASFs	5, 10 and 20 μM	Decreasing inflammation	IL-6↓, CXCL8↓, MMP-2↓, COX-2↓, TAK-1↓, p-p38↓, NF-κB↓	(95)
	Epigallocatechin	Tea	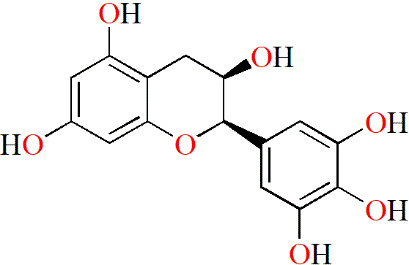		IL-1β–induced RASFs	5, 10 and 20 μM	Decreasing inflammation	IL-6↓, CXCL8↓, MMP-2↓, COX-2↓, TAK-1↓	(95)
Polysaccharides	*Lycium barbarum* L. polysaccharide	*Lycium barbarum* L.		CIA mice		25, 50, 100 mg/kg/d	Reducing paw thickness and CIA score; Attenuating joint damage; Decreasing inflammation	TNF↓, IL-6↓, IL-17↓, PGE2↓, MIP-1↓, anti-type II collagen IgG↓, MMP-1↓, MMP-3↓	(96)
Glycoside	Polyoxypregnane glycoside	*Dregea volubilis* (L.f) Benth. ex Hook. f	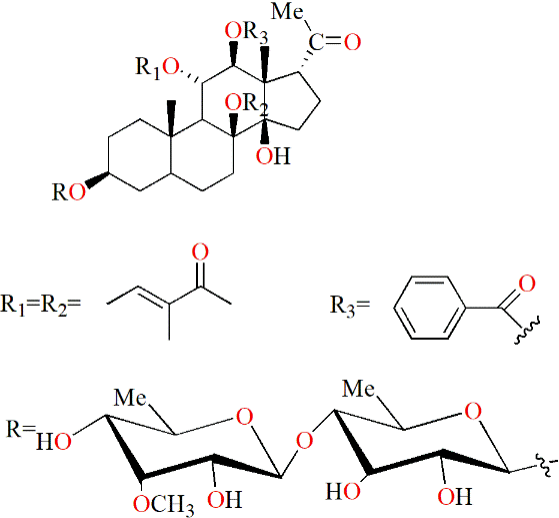		IL-1β–induced human articular chondrocyte (HAC)	6.25, 12.5 and 25 μM	Inhibiting cartilage degradation	MMP-1↓, MMP-3↓, MMP-13↓, IKKα/β↓, IκBα↑	(97)
	Cinnamomulactone	The dried twigs of *Cinnamomum cassia* (L.) J. Presl	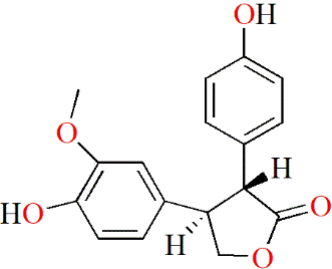		TNF-stimulated RASFs	0.1 μM		MMP-1↓, MMP-3↓, IL-1β↓	(98)
	(-)-Epicatechin-3-O-β-d-allopyranoside	*Davallia formosana* Hayata	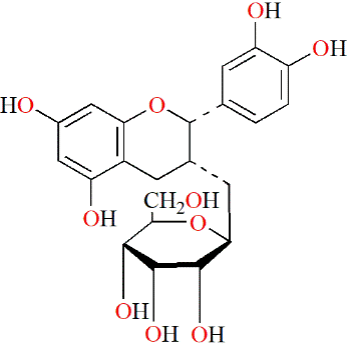	CIA mice		50 and 100 mg/kg/d	Suppressing the development of CIA; Decreasing inflammation	MMP-9↓, IL-1β↓, TNF↓, IL-17↓, IL-10↑, IL-4↑, IgG1↓, IgG2a↓, CD4^+^CD25^+^ regulatory T cells↓	(99)

### 5.2 Monomers from herbal medicines

#### 5.2.1 Terpenoids

Triptolide is a diterpenoid triepoxide that is extracted from the traditional Chinese herbal *Tripterygium wilfordii* Hook. f. (TWHF), which has been reported to have therapeutic effects on RA. Lin et al. initially explored the molecular mechanisms of triptolide in treating RA, and found that triptolide effectively inhibited messenger RNA levels and production of proMMP-1 and -3 while inhibiting PGE2 production by suppressing COX-2 expressions in IL-1α-treated human synovial fibroblasts. In addition, triptolide decreased the levels of IL-1β and IL-6 in LPS-treated mouse macrophages. These results suggested that the anti-RA activity shown by triptolide is due at least in part to anti-inflammatory activity (91). In the experiments of Liacini et al., triptolide was found to inhibit the expressions of MMP-3 and MMP-13 in a variety of cartilage destruction models, such as primary human OA chondrocytes, SW1353 cells, bovine chondrocytes and human synovial fibroblasts stimulated by cytokines and human and bovine cartilage explants stimulated by IL-1. In addition, triptolide also inhibited ADAMTS-4 expressions in bovine chondrocytes induced by IL-1-, IL-17- and TNF. All of these results suggest that triptolide has the potential to protect cartilage (92). Other details are shown in **Table 3**.

Pristimerin is a natural triterpenoid product that is derived from the family *Celastraceae*. In Tong et al. ‘s experiment, pristimerin effectively inhibited the inflammatory response in arthritic rats by inhibiting the proinflammatory cytokines IL-6, IL-17, IL-18 and IL-23 and promoting the anti-inflammatory factor IL-10. Subsequently, decreases in IL-6 and IL-17 directly led to a decrease in MMP-9 activity, thus causing pristimerin to have a protective effect on articular cartilage and bone (93). However, whether pristimerin directly affects MMP-9 could not be confirmed in this study.

Swertiamarin, another terpenoid with therapeutic potential for RA, was derived from *Enicostema axillare* (Lam.) A. Raynal (Gentianaceae). When swertiamarin was used to treat arthritic rats, it significantly reduced rat paw thickness, inhibited synovial monocyte infiltration, and protected joint tissues. After treatment, the plasma levels of IL-1, TNF and IL-6 were decreased, which were accompanied by decreased mRNA levels of MMP-9, iNOS, PGE2, PPARγ and COX-2, while IL-10 and IL-4 were increased. Further exploration of the mechanism behind swertiamarin showed that swertiamarin could inhibit RA development by inhibiting the NF-κB/IκB and JAK2/STAT3 signaling pathways (94).

#### 5.2.2 Flavonoids

Green tea is a popular beverage worldwide, and it is clear that catechins are the most active ingredients in green tea. Fechtner et al. studied whether epigallocatechin-3-gallate (EGCG), epigallocatechin (EGC), and epicatechin (EC), three catechins in green tea, had anti-RA effects. Based on their results, EGCG and EGC suppress IL-6, CXCL8 and MMP-2 production, and selectively decrease the expression of COX-2 in IL-1β–treated RASFs. However, EC did not show the same effects as described above. Thus, GCG and EGC partly contributed to the anti-inflammatory effect of green tea (95).

#### 5.2.3 Polysaccharides

After treating CIA mice with *Lycium barbarum* L. polysaccharide (LBP), Liu et al. found that LBP could exert anti-RA effects by downregulating inflammatory mediators and inhibiting joint bone damage through MMPs. Specifically, LBP reversed abnormal increases in inflammatory factors such as TNF-, IL-6 and IL-17 in CIA mice, decreased the protein expressions of MMP-1 and MMP-3, alleviated the ankle swelling in CIA mice, and increased the bone volume (96).

#### 5.2.4 Glycosides

Polyoxypregnane glycoside (PPG), which was extracted from the roots of *Dregea volubilis* (L.f) Benth. ex Hook. f, may have therapeutic potential for RA. Itthiarbha et al. showed that PPG inhibited the expression of MMP-1, MMP-3 and MMP-13 by inhibiting NF-κB activation in IL-1β-treated human articular chondrocytes. In addition, they further examined the mRNA levels of MMP-1, -3 and -13, and concluded that PPG could directly downregulate MMP expression by reducing mRNA levels, thus inhibiting the degradation of type II collagen (97).

In addition, in Kim’s experiments, a new butyrolactone compound, cinnamomulactone, was isolated from *Cinnamomum cassia* (Lauraceae) for the first time. In a quantitative real-time PCR (qPCR) assay, cinnamomulactone was found to effectively reduce the gene expressions of MMP-1 and MMP-3 in TNF-stimulated synovial fibroblasts, suggesting a potential therapeutic effect on RA (98).

(−)-Epicatechin-3-O-β-D-allopyranoside (ECAP) is a glycoside isolated from *Davallia formosana*. ECAP significantly inhibited knee cartilage erosion and reduced arthritis scores in CIA model mice. In addition, ECAP also reduced the levels of TNF and IL-17, increased the levels of IL-10 and IL-4, and inhibited IL-1 and MMP-9 expressions in CIA mice (99).

## 6 Conclusion and perspectives

As mentioned above, although RA is a nonfatal disease, RA seriously threatens the quality of life of patients and may even reduce their life expectancy due to its non-negligible damage to the joints. Previous studies have confirmed that MMPs, as important proteolytic enzymes, are involved in multiple RA processes, in which articular cartilage matrix destruction is particularly important. Therefore, targeted regulation of MMPs has become a research hotspot in the prevention and treatment of RA.

Disappointingly, there are limitations to the existing therapeutic approaches that target MMPs. For example, some endogenous proteins such as alpha_2_-macroglobulin, which can block the activity of MMPs, are present in the plasma after being secreted by the liver. However, because this is a large tetrameric glycoprotein, it cannot cross blood vessels to enrich in the cartilage, limiting its action to the inflammatory fluid around the joint. At present, the development of MMP inhibitors is mainly focused on inhibiting the active effects of MMPs. Although much research has been devoted to small molecule inhibitors, single-target inhibitors have not yet been developed. Non-selective MMP inhibitors may decrease multiple MMPs, inhibit the low levels of MMPs required for the normal physiological turnover of connective tissue, and thus cause significant side effects in the organism. Therefore, the effects of targeted MMPs in treating RA have not reached expectations.

Due to the unique multitarget effects of herbal medicines, they have gradually attracted the attention of researchers. In recent years, an increasing number of studies have focused on RA treatment that regulate MMPs. In existing studies, researchers have attempted to explore the efficacy and underlying mechanisms of herbal medicine in RA treatment through in vivo and in vitro experiments. To a certain extent, they have found some drugs with therapeutic potential. After a relevant comprehensive analysis, it was found that there are also some problems that cannot be ignored. In this paper, we found that dozens of herbal medicines can exert therapeutic effects on RA. Among these, some studies have confirmed that herbal medicines can directly inhibit collagen or proteoglycan degradation by affecting MMPs, thereby protecting joint structure. Remaining studies have focused more on alleviating the development and progression of RA by suppressing inflammation. In addition, some studies have shown that herbal medicines can protect joint structure, but this protection is only an indirect result after inhibiting inflammation, rather than directly protecting joint structure through MMPs. In addition, some MMPs, including MMP-3 and MMP-13, have been suggested for use as serum markers for RA diagnosis. Therefore, in these studies, MMPs were used only as biomarkers to compare their changes before and after treatment to determine the effects of herbal medicines. However, in this process, some researchers have ignored the specific roles of MMPs in RA, so they have not further explored the manner in through which MMPs are regulated by herbal medicines in experiments. In addition, although these herbal medicines have been experimentally validated in vitro and in vivo, most of them remain in preclinical research, and only a few herbs have been tested in clinical trials. More work needs to be done before herbal medicines can actually be applied in clinical trials. Therefore, this review only provides a reference for the majority of researchers, so that will have more possibilities for treating RA.

## Author contributions

All authors contributed to the article and approved the submitted version. WP, S-QL and C-JW conceived this paper. R-LL, H-XD and QL took part in drafting, revising and critically reviewing the article. YH and QZ gave final approval of the version to be published. WP, S-QL and C-JW have agreed on the journal to which the article has been submitted and agree to be accountable for all aspects of the work.

## Funding

This work was supported by the Project of Administration of Traditional Chinese Medicine of Sichuan Province of China (No. 2021MS407, 2020HJZX001), Special Research Project of Sichuan Traditional Chinese Medicine Administration (No.2021MS220) and Research Promotion Plan for Xinglin Scholars in Chengdu University of Traditional Chinese Medicine (No. QNXZ2019018) and the Natural Science Foundation of Sichuan Province (No. 2022NSFSC0720).

## Conflict of interest

The authors declare that the research was conducted in the absence of any commercial or financial relationships that could be construed as a potential conflict of interest.

## Publisher’s note

All claims expressed in this article are solely those of the authors and do not necessarily represent those of their affiliated organizations, or those of the publisher, the editors and the reviewers. Any product that may be evaluated in this article, or claim that may be made by its manufacturer, is not guaranteed or endorsed by the publisher.

## Glossary

**Table:**

ACR	aqueous extract of Cinnamomi ramulus
ADAM	a disintegrin and a metalloproteinase
ADAMTS	a disintegrin and metalloproteinase with thrombospondin type 1 motif
AP-1	activator protein 1
AS	Root of Angelica sinensis (Oliv.) Diels
ASLL	alkaloid extract from SL without lappaconitine
bFGF	basic fibroblast growth factor
CIA	collagen-induced arthritis
CLO	Celastrus orbiculatus Thunb.
CRT	compound Ruteng
DMARDs	disease-modifying anti-rheumatic drugs;
EAAS	ethyl acetate fraction from Angelica sinensis var. sinensis
EC	epicatechin
ECAP	(−)-epicatechin-3-O-b-D-allopyranoside
EGC	epigallocatechin
EGCG	epigallocatechin-3-gallate
EGF	epidermal growth factor
ESP	extract of Salvia plebeia R. Br.
EVOO	extra virgin olive oil
FCA	Freund’s complete adjuvant
FST	Fufang Shatai Heji
GAGs	glycosaminoglycans
GE	Gastrodia elata Blume
GPI	glycosylphosphatidylinositol
Hpx	hemopexin
HS	heterogeneous subgroups
HSR	hot-water extracts of Salacia reticulata Wight
JR	Jeevaneeya rasayana
LBP	Lycium barbarum L. polysaccharide
MCP-3	monocyte chemoattractant protein-3
MMPs	matrix metalloproteinases;
MT	membrane-type
NSAIDs	nonsteroidal anti-inflammatory drugs;
PDGF	platelet-derived growth factor
PE	polyphenolic extract
PEA3	polyomavirus enhancer activator-3
PPG	polyoxypregnane glycoside
QG	quercetin-3ß-D
QLY	Qing-Luo-Yin
qPCR	quantitative real-time PCR
RA	rheumatoid arthritis
RASF	RA synovial fibroblasts
SL	Strychnos spuxvomica L.
SP	signal peptide
SS	Stachys inflata var. caucasica Stschegl
TACE	TNF-a converting enzyme
TBL	Tongbiling
TGF-b	transforming growth factor b
TIMPs	tissue inhibitors of MMPs
TWHF	Tripterygium wilfordii Hook. F.
